# Sodium butyrate aids brain injury repair in neonatal rats

**DOI:** 10.1515/biol-2022-1046

**Published:** 2025-04-25

**Authors:** Jing Zhao, Jun Zhang, Can Yang, Linlin Yin, Li Hou, Lin Jiang

**Affiliations:** Department of Neonatology, Affiliated Hospital of North Sichuan Medical College, No. 1, Mao Yuan South Road, Shunqing District, Nanchong, Sichuan, 637000, China; Department of Neonatology, Affiliated Hospital of North Sichuan Medical College, Nanchong, Sichuan, 637000, China

**Keywords:** sodium butyrate, gut microbiota, gut-brain axis, neonatal rats, brain injury

## Abstract

The aim of this study is to investigate the effects and mechanism of action of sodium butyrate (SB) on brain injury repair in neonatal rats. 126 neonatal SD rats were randomly allocated to 7 groups, and necrotizing enterocolitis (NEC) and hypoxic–ischemic brain injury (HIBI) rat models were established. Hematoxylin and eosin staining showed that SB intervention alleviated intestinal and brain injuries in the HIBI + SB, NEC + SB, and NEC + HIBI + SB groups. Compared to the NEC and NEC + HIBI groups, the NEC + SB and NEC + HIBI + SB groups had significantly higher interleukin (IL)-10 and lower IL-17 levels (*P* < 0.05). Immunohistochemistry revealed increased Bcl-2 expression and decreased Bax expression in the NEC + SB and NEC + HIBI + SB groups compared to the NEC and NEC + HIBI groups in intestinal and brain tissues (*P* < 0.05). Compared to the control group (CG), gut microbiota diversity decreased in the HIBI, NEC, and NEC + HIBI groups, and increased significantly in the HIBI + SB, NEC + SB, and NEC + HIBI + SB groups. SB may alleviate brain injury by modulating gut microbiota, affecting IL-10 and IL-17 levels, and regulating Bcl-2 and Bax expression in intestinal and brain tissues.

## Graphical abstract

**Figure:**
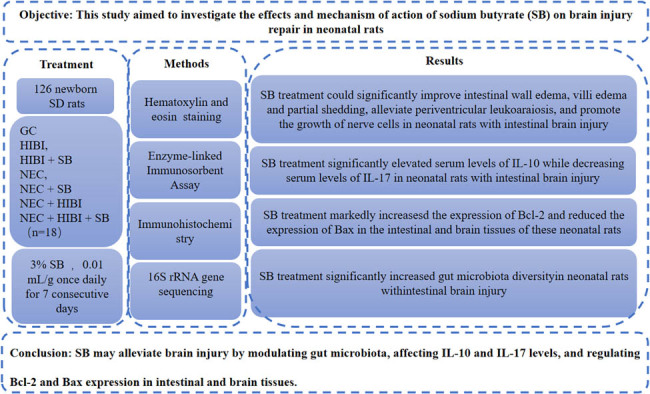

## 1 Introduction

Brain injury in premature infants results from brain ischemia or hemorrhagic damage caused by various pathological factors during prenatal or postnatal periods. In severe cases, it can lead to death [1,2]. Hypoxic-ischemia and inflammation are major contributors that lead to adverse neurodevelopmental outcomes in premature infants [3,4]. The global incidence of premature birth is approximately 11%. Premature infants have a significantly higher risk of developing cerebral palsy than full-term infants, and the risk of severe medical disabilities increases significantly as gestational age decreases [5,6]. Current diagnostic and treatment options for brain damage in premature infants are limited in clinical practice [7]. Therefore, identifying reasonable treatment options is crucial for clinical prevention and treatment of brain damage in premature infants.

Emerging evidence shows that early life gut microbiota plays a key role in brain development, communicating bidirectionally with the gastrointestinal system and the brain through neural, immune, and endocrine pathways [8]. The gut, gut microbiota, and brain interaction involves the release of various metabolites, including short-chain fatty acids (SCFAs), proteins, branched-chain fatty acids, and tryptophan metabolites [9,10]. Among these, SCFAs are organic monocarboxylic acids with a chain length of no more than six carbon atoms, mainly including butyrate, acetate, and propionate [11]. Sodium butyrate (SB), produced by gut microbiota, modulates gut microbiota, inhibits inflammatory factors, protects the intestinal mucosal barrier, and maintains gut immune balance [12,13,14]. Clinical studies suggest SB intervention improves ischemic stroke, reducing lipopolysaccharide and pro-inflammatory cytokines in the serum while enhancing the blood–brain barrier [15,16]. However, specific mechanisms underlying SB repair in premature infants remain unclear.

This study explores SB’s mechanism in repairing brain injury in premature infants using hypoxic–ischemic brain injury (HIBI) and necrotizing enterocolitis (NEC) rat models providing insights for potential treatment options.

## 2 Materials and methods

### 2.1 Animals

Pregnant Sprague-Dawley (SD) rats (SPF grade) about to give birth were provided by the Experimental Animal Center of North Sichuan Medical College and placed in a 12 h of alternating light and dark cycle, with free access to water and food. The birth status of pregnant rats was observed every 2 h, and newborn SD rats were allowed to consume breast milk freely. A total of 126 newborn SD rats (weighing 5–7 g) were randomly divided into seven groups: HIBI, HIBI + SB, NEC, NEC + SB, NEC + HIBI, NEC + HIBI + SB, and control group (CG), with 18 rats per group.


**Ethical approval:** The research related to animal use has been complied with all the relevant national regulations and institutional policies for the care and use of animals, and has been approved by the Animal Ethics Committee of the North Sichuan Medical College (approval number: 2021-135).

### 2.2 Establishment of NEC and HIBI models

Newborn SD rats were orally administered 0.1 mL of 3% sodium gluconate sulfate every 6 h [17] four times a day for 3 consecutive days to induce the NEC [18]. Additionally, 3-days-old SD rats were anesthetized with isoflurane, and the left common carotid artery was ligated and severed for 30 min. The rats were then housed in a hypoxic chamber (8% oxygen and 92% nitrogen) for 3 h to induce HIBI [19].

### 2.3 SB treatment

Following successful modeling at 3 days of age, rats in the HIBI + SB, NEC + SB, and NEC + HIBI + SB groups were orally administered 0.01 mL/g of 3% SB once daily for 7 consecutive days (303410-5g, Merck KgaA, Darmstadt, Germany) at 4 days of age. Serum, brain, and intestinal tissues, as well as intestinal fecal samples, were collected at 5 days of age. The brain and intestinal tissues, along with intestinal fecal samples, were collected at 10 days of age, and fecal samples were collected at 28 days of age.

### 2.4 Hematoxylin and eosin (HE) staining

Intestinal and brain tissues were collected from three rats in each group at 10 days old and stored in 4% paraformaldehyde [20]. The tissues were dehydrated and embedded in wax, and sectioned into 5 μm thin layers using a paraffin microtome. Sections were deparaffinized, dehydrated, and stained with HE. After removing excess staining solution, the tissues were rapidly dehydrated with ethanol, treated with xylene for transparency, and sealed with neutral resin before observation under a light microscope. Three fields of view were randomly selected from each specimen for analysis and photography. The histopathological changes of intestinal and brain tissues were studied at 400× magnification under a light microscopy (BA410, McAudi Industrial Group Co., Ltd, Fujian, China).

### 2.5 Enzyme-linked immunosorbent assay (ELISA)

Blood samples were obtained from the carotid arteries of 21 5-days-old rats, with three rats in each group. Whole blood was allowed to remain at 25°C for 15 min before being centrifuged at 3,000 rpm for 15 min to isolate serum. Interleukin (IL)-10 and IL-17 levels were detected using ELISA. The Rat IL-10 ELISA Kit (2R-KMLJr30194, Nanjing Camillo Biotechnology Company, Jiangsu, China), and Rat IL-17 ELISA Kit (2R-KMLJr30201, Nanjing Camillo Biotechnology Company, Jiangsu, China) were used to analyze the respective levels of IL-10 and IL-17.

### 2.6 Immunohistochemistry

The expression of Bcl-2 and Bax in intestinal and brain tissues was explored as described previously [21]. Intestinal and brain tissues were collected from three rats from each group at 5 days of age. Tissues were dehydrated, embedded in wax, and sectioned. Subsequently, the samples were dewaxed with xylene and hydrated using a series of ethanol solutions at varying concentrations (100, 95, 85, and 75%). Subsequently, cells were heated in citrate buffer for 20 min to facilitate antigen retrieval. The sections were heated and incubated for 10 min with 3% hydrogen peroxide, followed by blocking with goat serum for 20 min. Afterward, they were incubated overnight at 4°C with RabbitAnti-Bcl-2 (Bs-20352R, Beijing Boorsen Biotechnology Co., Ltd, Beijing, China) and Rabbit Anti-Bax (Bs-0127R, Beijing Boorsen Biotechnology Co., Ltd, Beijing, China). Sections were heated and incubated with BIOTIN-labeled Goat Anti-Rabbit IgG (SP9001, Beijing Zhongshan Jinqiao Biotechnology Co., Ltd, Beijing, China) at 37°C for 30 min. 3,3'-Diaminobenzidine (DAB) was used for color development, followed by a 3 min counterstaining of the nuclei with hematoxylin. The cells were then dehydrated, made transparent, and sealed with neutral gum. Images were captured at 400× magnification using a microscope (IX73; Olympus, Tokyo, Japan).

### 2.7 16S rRNA gene sequencing

Fecal samples were collected from newborn rats in each group at 5, 10, and 28 days of age (*n* = 3). The QIAamp Power Fecal QIAcube HT kit (OMEGA-soilDNAKit, TransGen Biotech, Beijing, China) was used to extract genomic DNA from fecal samples. Primers (341F: 5′-CCTAYGGGRBGCASCAG-3′, 806R: 5′-GGACTACNNGGGTATCTAAT-3′) were used to amplify bacterial DNA targeting V3 + V4 regions. DNA was sequenced using a NovaSeq 6000 PE250 platform. The original sequence was divided using the QIIME2demux plugin after generating high-quality sequences. The QIIME2 data plugin was used to control, trim, denoise, splice, and remove chimeras from the split sequence, to obtain clean reads. The resulting clean reads underwent processing that include the removal of the 341F/806R primer pair and clustering to create operational taxonomic units (OTUs) using the Vsearch software, at a similarity threshold of 97%. Representative reads for each OTU were selected using the QIIME package, and all representative sequences were compared and annotated using a database. The Green genes database was used for comparison, and the species were annotated using RDP classifier software. The R package vegan (https://github.com/vegandevs/vegan) was utilized to calculate alpha and beta. Alpha diversity is represented by the Shannon and Richness indices, where the Shannon index reflects the number and distribution of microbial species in a sample and richness represents the number of different species. A principal coordinate analysis (PCoA) of Bray-Curtis distances was performed to assess beta diversity.

### 2.8 Statistical analysis

Statistical analysis was conducted using SPSS 25.0, with data presented as mean value ± SD. Multiple comparisons between groups were conducted using single factor analysis of variance (Dunnett’s correction for multiple comparison), and the LSD method used for pairwise comparisons. Similarity analysis of gut microbiota was conducted using non-parametric tests. *P* < 0.05 indicates a statistically significance.

## 3 Results

### 3.1 SB ameliorated pathological intestinal and brain injury in neonatal rats

**Figure 1:**
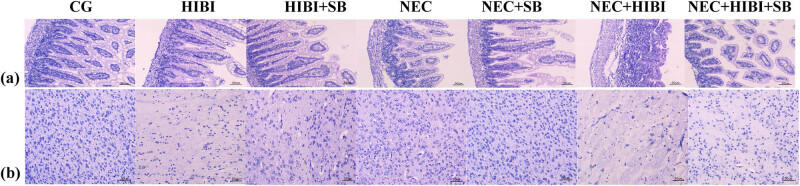
SB ameliorated pathological intestinal and brain injury in neonatal rats. (a) HE staining of intestinal tissue. (b) HE staining of brain tissue.

As shown in Figure 1a, the HIBI group exhibited villous edema and partial shedding, whereas the NEC group demonstrated edema of the intestinal wall, necrosis and shedding of the villi, and separation of the submucosal layer from the lamina propria. The NEC + HIBI group displayed intestinal wall edema, separation of the submucosal layer from the lamina propria, rupture of the muscle layer, extensive shedding of intestinal epithelial cells, and disappearance of villi. Compared to the model group, the HIBI + SB, NEC + SB, and NEC + HIBI + SB groups showed significant improvements in pathological manifestations. Additionally, the HE staining revealed pathological brain injury in rats. The number of nerve cells in the HIBI group was remarkably reduced, and the white matter around the ventricles appeared loose. In the NEC group, the nerve cell count decreased, with some nerve cells swelling to varying degrees, leading to a disordered arrangement, widened gaps, and loose white matter around the ventricles. Neurons in the NEC + HIBI group were sparse with nuclear pyknosis, severe white matter looseness around the ventricles, and reticular softening lesions. Again, compared to the model group, the HIBI + SB, NEC + SB, and NEC + HIBI + SB groups displayed significant improvements in these pathological manifestations. These findings demonstrate that SB ameliorated pathological intestinal and brain injury in neonatal rats (Figure 1b).

### 3.2 SB regulated the expression of IL-10 and IL-17

**Figure 2:**
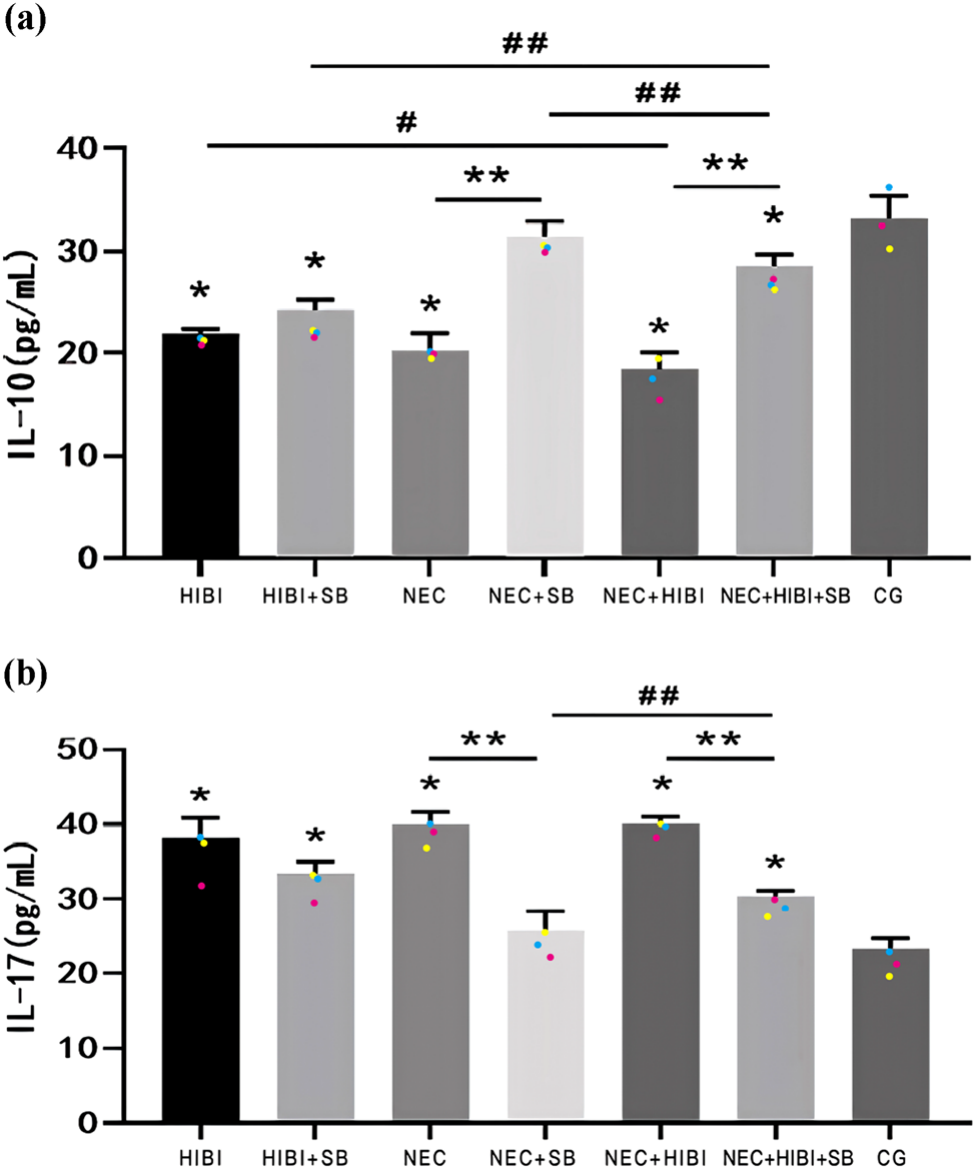
SB regulated the expression of IL-10 and IL-17. (a) ELISA results of IL-10. (b) ELISA results of IL-17. *P < 0.05, **P < 0.01; ^#^ *P* < 0.05, ^##^ *P* < 0.01.

IL-10 and IL-17 levels were measured by ELISA (Figure 2). When compared to the HIBI, NEC, and NEC + HIBI groups, IL-10 levels in the HIBI + SB, NEC + SB, and NEC + HIBI + SB groups increased remarkably (*P* < 0.05; Figure 2a). Conversely, IL-17 levels in the NEC + SB and NEC + HIBI + SB groups were significantly lower than those in the NEC and NEC + HIBI groups (*P* < 0.05; Figure 2b).

### 3.3 Effect of SB on protein expression level of Bcl-2 and Bax in intestinal and brain tissues

**Figure 3:**
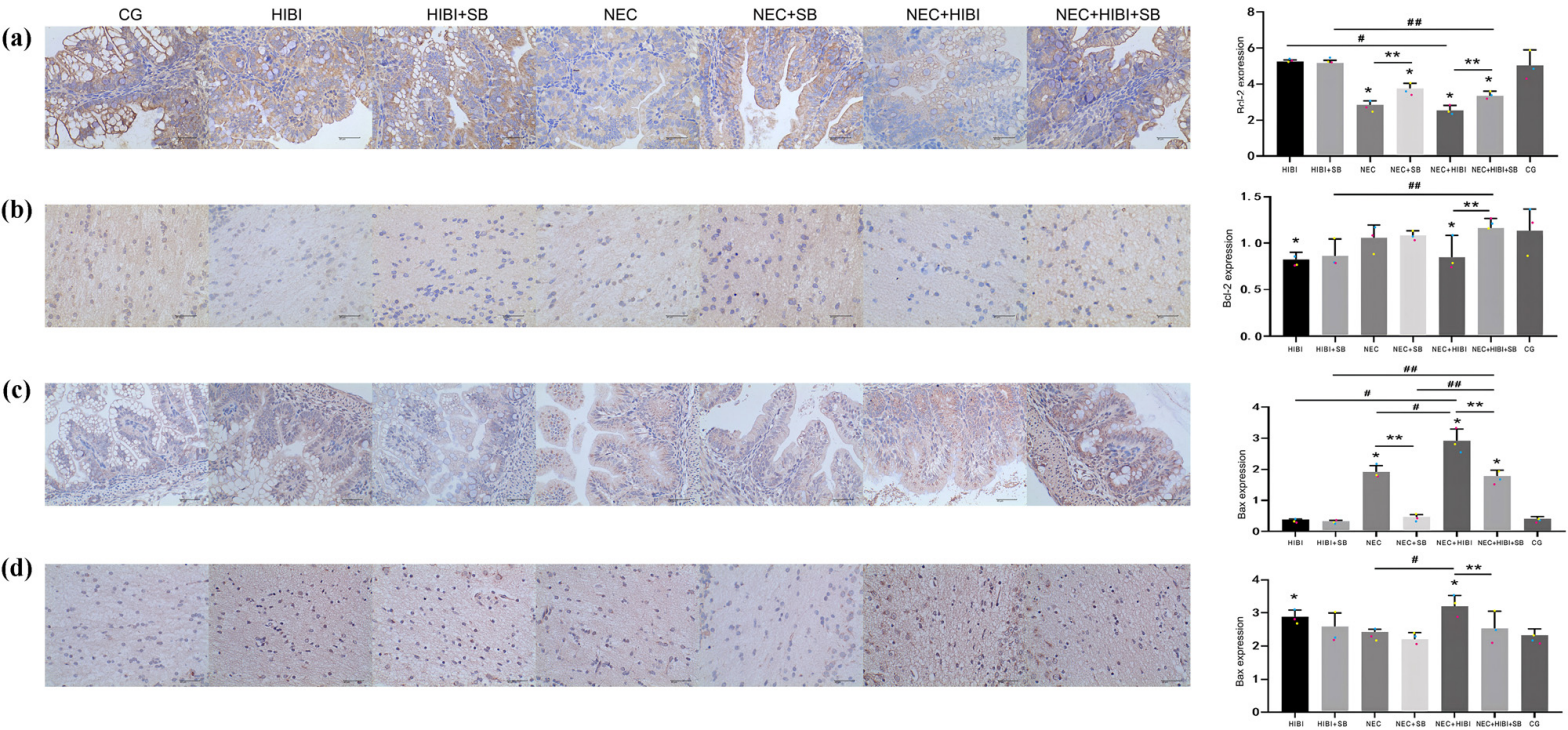
SB regulated the protein expression of Bcl-2 and Bax in intestinal and brain tissues. Immunohistochemistry result of Bcl-2 protein expression in intestinal (a) and brain (b) tissues. Immunohistochemistry result of Bax protein expression in intestinal (c) and brain (d) tissues. *P < 0.05, **P < 0.01; ^#^ *P* < 0.05, ^##^ *P* < 0.01.

In the intestinal tissue, Bcl-2 protein expression in the NEC + HIBI group was remarkably lower than that in the HIBI group (*P* < 0.05; Figure 3a). However, Bcl-2 protein expression increased significantly following SB treatment in the NEC and NEC + HIBI groups (*P* < 0.05; Figure 3a). Similarly, the Bcl-2 protein level in the brain tissue was increased in the NEC + HIBI + SB group compared to that in the NEC + HIBI group (*P* < 0.01; Figure 3b). In addition, in the intestinal tissue, the NEC + HIBI group showed a significant increase in Bax protein expression compared to the HIBI group (*P* < 0.05; Figure 3c). Conversely, Bax protein expression decreased significantly following SB treatment in the NEC and NEC + HIBI groups (*P* < 0.01; Figure 3c), aligning with the trend observed in brain tissue (Figure 3d).

### 3.4 Effect of SB treatment on the gut microbiota

**Figure 4:**
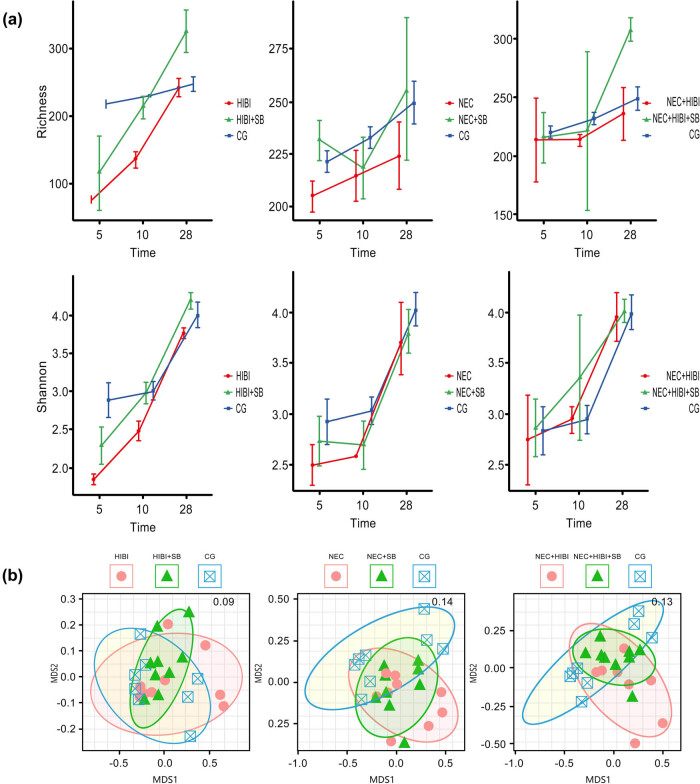
Effect of SB treatment on the gut microbiota. (a) Alpha diversity results. (b) PCoA.

Alterations in the gut microbiota were assessed using 16S rRNA gene sequencing. The alpha diversity results indicated a reduction in microbial diversity in the HIBI, NEC, and NEC + HIBI groups compared to the CG group, whereas microbial diversity was increased after SB treatment in the HIBI, NEC, and NEC + HIBI groups (Figure 4a). Moreover, PCA of the gut microbiota showed remarkable differences between the HIBI, NEC, and NEC + HIBI groups compared to the CG group, with samples from the HIBI + SB, NEC + SB, and NEC + HIBI + SB groups tending to be closer to the CG group (Figure 4b). Statistical analysis of microbial community similarity revealed no significant difference between the HIBI and HIBI + SB groups compared to the CG group; however, significant differences were observed in the NEC, NEC + SB, NEC + HIBI, and NEC + HIBI + SB groups (*P* < 0.05; Table 1).

**Table 1:** Statistical analysis of microbial community similarity

Group	*R* ^2^	*P*
HIBI, HIBI + SB, CG	0.116	0.104
NEC, NEC + SB, CG	0.142	0.023*
NEC + HIBI, NEC + HIBI + SB, CG	0.166	0.006*

Note: *P < 0.05, compared to CG group.

## 4 Discussion

SB, a histone deacetylase inhibitor, has been reported to provide neuroprotection to neonatal rats with HIBI [22,23]. Ziemka-Nalecz et al. showed that SB demonstrated neuroprotective and neurogenic effects in a rat model of neonatal hypoxia-ischemia [24]. Moreover, Sun et al. revealed that SB mitigated intestinal inflammation in mice with NEC [25]. Consistent with these findings, this study demonstrated that SB intervention significantly elevated the Bcl-2/Bax ratio in all model rats, enhanced the secretion of the IL-10 cytokine, reduced the levels of the pro-inflammatory IL-17 factor, increased the α diversity of intestinal microbiota, and consequently mitigated neuronal cell apoptosis and inflammation. These effects led to improved pathological outcomes in both the intestinal tract and brain of neonatal rats with injuries.

Cytokines, including IL-10 and IL-17, play a crucial role in regulating the inflammatory response and tissue damage within the immune system. These cytokines are also implicated in the progression and treatment of various neurodegenerative and autoimmune diseases. Studies have demonstrated that Treg and γδT cells are activated and migrate to the damaged area following a ischemic brain injury, where they rapidly secrete IL-10 and IL-17, respectively [26,27]. IL-17 can activate glial cells, resulting in the release of pro-inflammatory factors such as TNF-α, which exacerbates brain tissue inflammation [28]. In contrast, IL-10 plays a crucial role in stimulating the repair response of microglia and in promoting neuronal survival [14]. Treg cells also secrete IL-10 to inhibit the matrix metalloproteinase (MMP-9) activity, thereby preventing the invasion of the blood–brain barrier by inflammatory cells [29]. In the late acute phase of ischemic brain injury, Treg cells were found in ischemic tissues, secreting large amounts of IL-10, which plays a neuroprotective role [30]. Research has indicated that the administration of butyrate elevates the levels of peripheral blood Treg cells and increases the plasma concentrations of anti-Th17 cytokines (IL-10 and IL-12). Additionally, butyrate treatment reduced IL-17 levels in both plasma and colonic mucosa, while improving lesions associated with colonic colitis in rats [31]. In this study, IL-10 levels increased and IL-17 levels decreased after SB treatment in neonatal rats, indicating that SB might have a protective effect on nerves by regulating inflammatory factors in neonatal rats.

The occurrence of brain injury in premature infants is typically concomitant with neuronal apoptosis [32]. Zhu et al. reported that more than 50% of degenerated cells in several brain regions of newborn rats after hypoxia-ischemia were apoptosis [33]. Bcl-2, a protein with anti-apoptotic properties, governs the process of apoptosis by engaging in direct or indirect interactions with the pro-apoptotic protein Bax. In this study, compared to the CG group, Bcl-2 expression reduced and Bax expression increased in the HIBI and NEC + HIBI groups, indicating that the imbalance of Bcl-2 and Bax expression was involved in neonatal rat brain injury. Further research found that after SB intervention, Bcl-2 expression increased and Bax expression decreased in the intestinal tissue. These results suggest that SB alleviates neuronal apoptosis and reduces brain injury in neonatal rats by modulating the expression of Bcl-2 and Bax.

Changes in gut microbiota are often accompanied by bidirectional communication between the intestine and the brain. Dysbiosis of the gut microbiota not only leads to gastrointestinal diseases, but also affects the nervous system [34]. Mohajeri et al. demonstrated that after repeated brain trauma in animals, the composition of gut microbiota was assessed at 6 h, 48 h, and 30 days, revealing a significant reduction in diversity in brain-injured animals at all time points [35]. Long-term treatment with SB can alter the composition of the gut microbiota and improve pathological brain damage. He et al. used SB at 100 mg/kg to treat hypoxic–ischemic brain damage in rats for 2 weeks and found that SB improves the pathology associated with HIBI by modifying the gut microbiota and brain SCFA levels, which subsequently influence the expression of neurotrophic-related genes mediated by histone crotonylation [23]. In a study conducted by Wang et al., type 2 Diabetes Mellitus mice underwent a 4-week intervention with SB, resulting in alterations in the gut microbiota and a reduction in brain injury following stroke [15]. This indicates that prolonged treatment with SB has the potential to modify gut microbiota composition and mitigate brain damage associated with pathology. This study observed that, compared to the CG group, microbial diversity in the HIBI, NEC, and NEC + HIBI groups decreased, indicating that changes in the gut microbiota might influence this bidirectional communication between the brain and the intestine. However, microbial diversity was significantly increased after SB treatment in the HIBI, NEC, and NEC + HIBI groups, suggesting that the level of SB might be related to the composition of the gut microbiota and the abundance of major bacterial phyla. However, study revealed that the composition and abundance of the gut baseline microbiota can metabolize substrates in various ways, thereby influencing the production and utilization of butyrate [36]. Additionally, environmental factors and a high dietary fiber intake can impact intestinal microbial diversity, consequently affecting butyrate utilization [37,38]. Given the constraints of the study conditions, a thorough exploration of these factors’ impact on SB treatment was not conducted. Nevertheless, this research mitigated the interference of these confounding factors by establishing a control group, replicating the experiments, and standardizing the diet and environmental conditions for the neonatal rats.

Although this study investigated the potential mechanisms of SB treatment for brain injury in neonatal rats, it has some limitations. First, the study was conducted exclusively using samples from newborn rats. Second, it did not explore the effect of inflammatory factors on the central nervous system or their specific mechanisms of action in brain injury. Finally, this study did not delve into the molecular mechanisms involving specific gut microbes and metabolites in the SB treatment of brain injury. Therefore, in the future, we will expand the experimental sample size and employ metabolomic and proteomic methodologies to further elucidate the molecular mechanisms by which SB regulates microorganisms in the treatment of neonatal rat brain injury.

## 5 Conclusion

In conclusion, SB, a metabolite of the intestinal flora, may alleviate brain injury in neonatal rats by modulating the structure of the gut microbiota, affecting the levels of the inflammatory factors IL-10 and IL-17, modulating the expression of Bcl-2 and Bax in intestinal and brain tissues, and reducing neuronal cell apoptosis. This study confirmed that intestinal inflammation plays a role in the onset and progression of brain injury in neonatal rats, thereby furnishing a robust theoretical foundation for the therapeutic use of SB in addressing brain injury.
